# Supplementary material for: Survival of the simplest in microbial evolution
Source: Nat Commun. 2019 Jun 6;10:2472. doi: 10.1038/s41467-019-10413-8 (PMC6554311; doi:10.1038/s41467-019-10413-8)
Supplement: Supplementary file 1 — Supplementary Information [file 41467_2019_10413_MOESM1_ESM.pdf]

Supplementary Information

Supplementary Methods 1: Stochastic theory of phenotypic interference

In the main text, we derive the scaling relations of phenotypic interference, Equation (3) – Equation (9), using the evolution equation for quantitative traits, Equation (15), in its deterministic limit ($\chi = 0$). Here we show that the fully stochastic evolution equation generates the same scaling. These stochastic fluctuations generate an ensemble $Q(\Gamma, t)$, which is the distribution of Γ over parallel-evolving genes. We convert Equation (15) into an equivalent diffusion equation [1] for the probability density $Q(\Gamma, t)$,

$$\frac{\partial}{\partial t} Q(\Gamma, t) = \left[\tilde{\sigma} \Delta_G \frac{\partial^2}{\partial \Gamma^2} + \frac{\partial}{\partial \Gamma} \left(\kappa \epsilon_G u - \Delta_G f'(\Gamma) \right) \right] Q(\Gamma, t) \tag{S1}$$

with the average trait diversity Δ_G given by Equation (1). The equilibrium probability distribution $Q_{\rm eq}(\Gamma)$ describes the stationary fluctuations of the population mean trait $\Gamma(t)$ of a stable gene around its long-term average $\langle \Gamma \rangle = \int \Gamma \, Q_{\rm eq}(\Gamma) \, d\Gamma$; these fluctuations are generated by genetic drift and (predominantly) genetic draft. In the biophysical fitness landscape, Equation (13), the equilibrium distribution can be evaluated analytically,

$$Q_{\rm eq}(\Gamma) = \frac{\left(\frac{f_0}{\tilde{\sigma}} \right)^{2\kappa \frac{k_{\rm B}T}{\epsilon_G}} \exp\left(-\frac{2\kappa\Gamma}{\epsilon_G} - \frac{f_0}{\tilde{\sigma}} {\rm e}^{-\Gamma/k_{\rm B}T} \right)}{k_{\rm B}T \, {\rm Gamma} \left(2\kappa \frac{k_{\rm B}T}{\epsilon_G} \right)}, \tag{S2}$$

where Gamma and PolyGamma are standard transcendental functions. This function is plotted in Supplementary Figure 3a. The resulting average,

$$\frac{\langle \Gamma \rangle}{k_{\rm B}T} = -\log\left(\frac{\tilde{\sigma}}{f_0} \right) - {\rm PolyGamma} \left(2\kappa \frac{k_{\rm B}T}{\epsilon_G} \right), \tag{S3}$$

shows that genes are slightly more stable than estimated from the deterministic average derived in the main text, $\Gamma/k_{\rm B}T = -\log(2\kappa\tilde{\sigma}k_{\rm B}T/f_0\epsilon_G)$. Through the nonlinearity of the fitness landscape, the fluctuations of the mean trait Γ induce fluctuations of the conditional average fitness variance, $\langle \Delta_f \rangle(\Gamma) = \Delta_G f'^2(\Gamma)$. We obtain the equilibrium distribution

$$Q_{\rm eq}(\Delta_f) = {\rm Gamma}_{\rm gen} \left(\Delta_f; \; 2\kappa \frac{k_{\rm B}T}{\epsilon_G}, \frac{1}{2} u \tilde{\sigma} \frac{\epsilon_G^2}{(k_{\rm B}T)^2}, \frac{1}{2}, 0 \right), \tag{S4}$$

with $\text{Gamma}_{\text{gen}}$ denoting the generalized gamma distribution (Supplementary Figure 3b). The average fitness variance

$$\langle \Delta_f \rangle = 2\tilde{\sigma} u \kappa^2 \left(1 + \frac{\epsilon_G}{2\kappa k_{\rm B}T} \right) \tag{S5}$$

differs from its deterministic counterpart, Equation (3) by a prefactor of order 1. Similarly, the Γ fluctuations induce fluctuations of the interference load of individual genes,

$$Q_{\rm eq}(\mathcal{L}_{\rm gene}) = {\rm Gamma}_{\rm dist} \left(\mathcal{L}_{\rm gene}; \; 2\kappa \frac{k_{\rm B}T}{\epsilon_G}, \tilde{\sigma} \right) \tag{S6}$$

(Supplementary Figure 3c). The resulting dependence

$$\langle \mathcal{L}_{\rm gene} \rangle = 2\kappa \frac{k_{\rm B}T}{\epsilon_G} \tilde{\sigma} \tag{S7}$$

is identical to the deterministic case; the fluctuation effect on $\langle \Gamma \rangle$, Supplementary Equation S3, is offset by the fluctuation load in a downward-curved fitness landscape.

Supplementary Methods 2: Cross-over scaling of the fitness wave

An asexual mutation-selection-drift process with genomic sites of fixed selection coefficient s has two distinct scaling regimes [2, 3],

$$\sigma^2 = \begin{cases} sug, & (g \lesssim g_\mathrm{c}) \\ c\tilde{\sigma}^2 = (\frac{c}{4})^{1/3}(s^2 ug)^{2/3} & (g \gtrsim g_\mathrm{c}), \end{cases} \tag{S8}$$

which correspond to independently evolving sites and to an asymptotic fitness wave with strong interference selection, respectively. At the crossover point $g_\mathrm{c} = cs/(4u)$, the relation

$$\tilde{\sigma}(g_\mathrm{c}) = \frac{2ug_\mathrm{c}}{c} = \frac{1}{2}s \tag{S9}$$

is valid. Comparing this relation with the *generic* scaling under phenotypic interference, $\tilde{\sigma} = 2ug/c = s/2$ as given by Equation (5) and Equation (6), we conclude that the phenotypic fitness wave is locked in the crossover region of marginal interference.

This scaling has two important consequences. First, the phenotypic interference process has a sufficiently high mutational input for fitness wave theory to be applicable, $ug \gtrsim s$ [3,4]. Second, as discussed in the main text, Supplementary Equation S9 expresses the feedback between global and local selection in a phenotypic fitness landscape, which tunes selection coeffecents to the g-dependent value $s = 4ug/c$. Consistently, the phenotypic fitness wave has a fitness variance $\sigma^2 \sim g^2$, compared to the scaling $\sigma^2 \sim g^{4/3}$ of the asymptotic regime at fixed selection coefficients (up to log corrections).

Supplementary Methods 3: Extended biophysical fitness models

In this section, we develop alternative evolutionary models of quantitative traits under genetic linkage. The mode of phenotypic interference, which is characterized by a superlinear scaling of the genetic load with genome complexity, occurs in all cases, suggesting it is a generic property of this class of models. Specifically, we discuss housekeeping dynamics in extended models of protein evolution.

Active protein degradation. This non-equilibrium process affects a wide range of proteins, for example through the ubiquitin-proteasome pathway [5]. It ensures that regulatory proteins are rapidly cleared once their function ends (at a particular point of the cell cycle). Consider a simple model that has thermal rates of folding and unfolding, κ_G^+ and κ_G^-, given by

$$\kappa_G^\pm(G) = \frac{\kappa_G^0}{1 + \mathrm{e}^{\pm G/k_\mathrm{B}T}}. \tag{S10}$$

These rates satisfy the detailed balance condition $\kappa^-(G)/\kappa^+(G) = \exp(-G/k_\mathrm{B}T)$ and become kinetically limited in the regime of a large drop in free energy, $\kappa_G^\pm \simeq \kappa_G^0$ for $\pm G/k_\mathrm{B}T \gg 1$. Active protein degradation takes place with a rate $r \gg \kappa_G^-(G)$ that supersedes the thermal process. Here we do not model details of the pathways of protein synthesis from and degradation into amino acid constituents, which would only affect the total protein concentration but not their state probabilities. In the steady state of the active-degradation model, proteins are folded with probability

$$p_+(G) = \frac{1}{1 + \nu_G + \nu_G\,\mathrm{e}^{-G/k_\mathrm{B}T}} \qquad \text{(active degradation)}, \tag{S11}$$

where $\nu_G = r/\kappa_G^0$. Hence, this model retains the sigmoid form of the fitness landscape given in Equation (13) and shown in Box 1 (top) of the main text, and our evolutionary conclusions remain invariant.

Stability-affinity model. This model extends the minimal protein model discussed in the main text by explicitly including protein function, which is assumed to be mediated through binding to a molecular target. Proteins can be in three thermodynamic states: functional, i.e., folded and target-bound (++), folded and unbound (+−), and unfolded (−−). We assume that unfolded proteins cannot bind their target, which implies that the fourth state of unfolded proteins localized to their target (−+) is suppressed by the entropy loss of localization. We consider two different thermodynamic ensembles of these proteins.

(i) In thermodynamic equilibrium, the statistics of this ensemble is governed by two quantitative traits, which are defined as free energy differences: the fold stability $G \equiv G_{--} - G_{+-}$ and the reduced binding affinity $E \equiv G_{+-} - G_{++}$, which includes the entropy loss of localization and depends on the ligand concentration. The equilibrium state probabilities p_{++}, p_{--}, and p_{--} are given by Boltzmann statistics depending on the traits G and E; in particular,

$$p_{++}(G,E) = \frac{1}{1 + \mathrm{e}^{-E/k_\mathrm{B}T} + \mathrm{e}^{-(E+G)/k_\mathrm{B}T}} \qquad \text{(thermodynamic equilibrium).} \tag{S12}$$

Equilibrium models of this kind are well known in protein biophysics [6, 7], and have been used to build fitness landscapes [8, 9].

(ii) Active degradation is again a ubiquitous process that drives the thermodynamics out of equilibrium; this process is particularly relevant for target-bound proteins that would have a long lifetime at thermodynamic equilibrium. Here we assume protein folding (−−) → (+−) takes place with a thermal rate $\kappa_G^+(G)$ given by Supplementary Equation S10, protein degradation takes place with a single rate r for the processes (++) → (−−) and (+−) → (−−), and the (un-)binding dynamics takes place with thermal rates $\kappa_E^\pm(E)$ analogous to Supplementary Equation S10. In this model, the folding and binding processes decouple, and we obtain the non-equilibrium steady-state probability

$$p_{++}(G,E) = \frac{1}{1 + \nu_G + \nu_G \mathrm{e}^{-G/k_\mathrm{B}T}} \frac{1}{(1+\nu_E)(1 + \mathrm{e}^{-E/k_\mathrm{B}T})} \qquad \text{(active degradation)} \tag{S13}$$

with $\nu_G = r/\kappa_G^0$ and $\nu_E = r/\kappa_E^0$.

From these ensembles, we build thermodynamic fitness landscapes

$$f(G,E) = f_0\, p_{++}(G,E) \tag{S14}$$

analogous to Equation (13); these landscapes are plotted in Supplementary Figure 5ab.

The population genetics of the two-trait system is described by the population mean values Γ_G and Γ_E, the diversities Δ_{GG} and Δ_{EE}, and the covariance Δ_{GE}. Under mutations, coalescence, and selection given by the fitness landscape $f(G,E)$, the mean traits follow a stochastic evolution equation analogous to Equation (15),

$$\begin{pmatrix} \dot\Gamma_G \\ \dot\Gamma_E \end{pmatrix} = -\begin{pmatrix} u_G \kappa_G \epsilon_G \\ u_E \kappa_E \epsilon_E \end{pmatrix} + \begin{pmatrix} \Delta_{GG} & \Delta_{GE} \\ \Delta_{GE} & \Delta_{EE} \end{pmatrix} \begin{pmatrix} \partial_G f(E,G) \\ \partial_E f(E,G) \end{pmatrix} + \begin{pmatrix} \chi_G \\ \chi_E \end{pmatrix} \tag{S15}$$

with white noise of mean and variance

$$\begin{pmatrix} \langle \chi_G \rangle \\ \langle \chi_E \rangle \end{pmatrix} = \begin{pmatrix} 0 \\ 0 \end{pmatrix}, \qquad \begin{pmatrix} \langle \chi_G(t)\chi_G(t')\rangle & \langle \chi_G(t)\chi_E(t')\rangle \\ \langle \chi_G(t)\chi_E(t')\rangle & \langle \chi_E(t)\chi_E(t')\rangle \end{pmatrix} = \tilde\sigma \delta(t-t') \begin{pmatrix} \Delta_{GG} & \Delta_{GE} \\ \Delta_{GE} & \Delta_{EE} \end{pmatrix}. \tag{S16}$$

Here we discuss the simplest stationary states of housekeeping evolution in this model, using the deterministic limit of the evolution equation ($\chi_G = \chi_E = 0$). The trait diversities Δ_{GG} and Δ_{EE} are given as in Equation (1), and we assume that pleiotropic sites have uncorrelated effects on both traits, i.e., $\Delta_{GE} = 0$; this has recently been observed in [10]. As in the main text, we set $\kappa_G = \kappa_E = 1$, which says that most random mutations reduce stability and affinity.

In equilibrium, the high-fitness part of the fitness landscape takes the asymptotic form $f(G,E) \simeq f_0[1 - \mathrm{e}^{-E/k_\mathrm{B}T}(1 + \mathrm{e}^{-G/k_\mathrm{B}T} + \mathrm{e}^{-E/k_\mathrm{B}T})] + O((e^{-E/k_\mathrm{B}T}, e^{-G/k_\mathrm{B}T})^3)$. The mutation-selection equilibrium leads to

mean trait values

$$\begin{pmatrix}\Gamma_G \\ \Gamma_E\end{pmatrix} \approx \begin{pmatrix} k_{\rm B}T \log\left(\frac{\epsilon_G}{\epsilon_E} - 1\right) \\ -k_{\rm B}T \log\left(2\frac{\tilde{\sigma}}{f_0}\left(\frac{k_{\rm B}T}{\epsilon_E} - \frac{k_{\rm B}T}{\epsilon_G}\right)\right)\end{pmatrix}, \tag{S17}$$

where only the E-component depends on the coalescence rate $\tilde{\sigma}$. Comparison with the minimal model, Equation (3), shows that in the stable part of the fitness landscape, the equilibrium stability-affinity model becomes an essentially one-dimensional problem for the affinity trait E [8]. The total fitness variance per gene, $\Delta_f = 2(u_G + u_E)\tilde{\sigma}$, is of the universal form Equation (3) with an effective mutation rate

$$u = u_G + u_E. \tag{S18}$$

We conclude that housekeeping evolution in this model follows the same scaling as in the minimal model, Equation (3) – Equation (12), with the parameter u given by Supplementary Equation S18. However, the equilibrium model lacks evolutionary stability, because lack of folding stability ($G > 0$) can be compensated by a stronger binding affinity.

With active degradation, the high-fitness part of the fitness landscape takes the asymptotic form $f(G, E) \simeq f_0[1 - \mathrm{e}^{-E/k_{\rm B}T} - \nu_G/(1 + \nu_G)\,\mathrm{e}^{-G/k_{\rm B}T}] + O((e^{-E/k_{\rm B}T}, e^{-G/k_{\rm B}T})^2)$. Hence, for stable genes ($f_0 \gg \tilde{\sigma}$), the evolutionary dynamics of the traits G and E becomes approximately independent. The traits of each gene are at a mutation-selection equilibrium of the universal form Equation (3), generating a combined fitness variance $\Delta_f = 2(u_G + u_E)\tilde{\sigma}$. Therefore, housekeeping evolution in this model also follows the same scaling as in the minimal model, Equation (3) – Equation (12), with a total mutation rate per gene given by Supplementary Equation S18 and an effective value of g that is twice the number of genes,

$$g_{\rm eff} = 2g. \tag{S19}$$

In particular, the system-wide interference load is about twice the value of the minimal model, $\mathcal{L}_{\rm int} \approx 8ug^2/c$, as used in the main text. This estimate disregards the additional contribution from the enhanced total mutation rate, Supplementary Equation S18, which takes into account that $u_E \ll u_G$ for many binding domains.

The form invariance of housekeeping evolution in these models shows the robustness of the phenotypic interference mode. It also suggests that in more general contexts, we can define genomic complexity as the number of quantitative traits that evolve (approximately) independently; see the Discussion of the main text.

Single-peak fitness model. A minimal model of stabilizing selection is a quadratic landscape [11–13],

$$f(E) = -f_0(E - E^*)^2 \tag{S20}$$

(Supplementary Figure 5c). This model penalizes deviations from an optimal trait value E^*. In contrast to the biophysical landscape, there is no gene loss in a quadratic landscape, because there are no constraints on its slope. As long as mutations generate trait equilibria predominantly on one flank of the landscape, the basic scaling of phenotypic interference, Equation (3) – Equation (7), is universal and, hence, the same as in the minimal model. The genetic load for a single gene, $\mathcal{L}_{\rm gene} = -f(\Gamma) = u^2\epsilon_E^2/4\Delta_E^2 f_0$, has been derived in [1]. With Δ_E given by Equation (1), we find a system-wide interference load

$$\mathcal{L}_{\rm int} = g\mathcal{L}_{\rm gene} = \frac{4u^2g^3}{c^2\epsilon_E^2 f_0}. \tag{S21}$$

Hence, the single-peak model has an even stronger load nonlinearity than the biophysical fitness landscapes.

Supplementary Discussion: Comparison of models for the evolution of recombination

A variety of models and hypotheses describe selective pathways for the evolution of sexual reproduction; see, e.g. refs. [14–16]. A feasible pathway has to be selected for, or at least not selected against, in the short-term and provide a net benefit of recombination in the long term. The phenotypic interference pathway discussed in the main text realizes these criteria in a two-stage process. Genetic changes enabling recombination at a rate R of order $R^* \sim ug$ involve a cost of order

$$\mathcal{L}_{\rm rec} \sim R^*; \tag{S22}$$

the subsequent elimination of the interference load generates a g-fold higher benefit of order

$$\mathcal{L}_{\rm int} \sim gR^*. \tag{S23}$$

We can compare this pathway with four broad classes of previous models:

- *Direct benefits* of recombination have been proposed in the literature; see, e.g., the reviews and collections [14–20]. For example, offspring from two parents can have an increased its survival chance, the recombination process can go along with a repair of damaged DNA or a reduced deleterious mutation rate, or selection becomes more efficient with mate choice. These models are not about breaking linkage correlations, and it has been shown that their effect is marginal in the evolutionary process [15, 16]. Moreover, the proposed mechanisms involve complex machinery and two sexes, so they do not apply to microbial evolution. Their short- and long-term benefit is at most linear in R and, hence, much smaller than the quadratic benefit $\mathcal{L}_{\rm int}$ in the phenotypic interference model.

- *Broad epistasis across genes* provides another selective benefit of recombination. For example, the mutational deterministic hypothesis posits negative epistasis between deleterious mutations across the entire genome [15, 21]. In a mutation-selection balance, this type of epistasis can halt the dynamics of Muller's ratchet at a mutational load $\mathcal{L} \sim U$ [22], where $U \gtrsim ug$ is the total genome-wide rate of deleterious mutations. In this model, recombination generates a a short-term benefit by breaking negative epistatic links, which leads to a long-term benefit $\Delta\mathcal{L} \sim U$ by elimination of the mutational load [15,16,21]. Again, this benefit scales linearly with g and, hence, is much smaller than the quadratic benefit in the phenotypic interference model. Moreover, the assumption of genome-wide epistasis is unrealistic as a generic selective pathway to recombination [16, 19]. In another model with broad, heterogeneous pairwise epistasis, recombination generates a transition from genotype selection to gene selection [23]. This causes a short-term benefit, but the transition causes a net fitness loss by breaking linkage between epistatic sites [23]. Our model replaces the assumption of global epistasis across the genome by local diminishing-return epistasis between loci affecting the same protein stability or affinity trait. This form of epistasis follows directly from the underlying thermodynamic nonlinearities, and its evolutionary effects differ from global epistasis.

- Models of *adaptive evolution* generate a benefit of recombination by enhancing adaptability to time-dependent selection pressures. An example is the red queen race of a host co-evolving with a parasite under epistasis generated by the host-parasite interaction [14, 16, 17, 20]. In these dynamics, recombination breaks linkage disequilibria and can generate rare, fitter individuals, which entails a "delayed-short term benefit" [24]. Although the red queen scenario has experimental evidence for specific systems [16, 18, 20], this type of models requires strong, continual adaptation, as well as stringent assumptions on the form of selection [16, 18, 20]. The phenotypic interference model requires none of these assumptions. The benefit of recombination arises already in a conservative and non-adaptive scenario of housekeeping evolution, and it depends only on local epistasis of individual protein traits. Moreover, a short-term benefit of recombination is not required because the cost $\mathcal{L}_{\rm rec}$ is marginal (i.e., of order of the inverse effective population size).

- Recent models of *interference selection* [3, 25–28] use a mutational input with rate U and a prescribed distribution of additive selective effects with mean f_0. The clonal interference dynamics in these models has two important quantitative differences to phenotypic interference. First, in models of predominantly deleterious mutations, the nonlinear effects of interference, which are associated with Muller's ratchet, set in at a rate $U_{\rm m} = f_0 \log(N_{\rm e} f_0)$ [25–27, 29, 30]. This corresponds to an onset gene number $g_{\rm m} = (f_0/u) \log(N_{\rm e} f_0)$ that is larger than the onset of phenotypic interference at $g_0 \sim c$ (Fig. 1b compares these scales for a reasonable selective effect strength of $f_0 = 200u$). Second, in models of adaptive evolution, the crossover from clonal to sexual evolution takes place at a characteristic recombination rate $\tilde{R} \sim \sqrt{N_{\rm e} U_{\rm b} f_0^2}$, where $U_{\rm b}$ denotes the genome-wide rate of beneficial mutations [3, 28]. Inserting the condition of clonal interference between beneficial mutations, $N_{\rm e} U_{\rm b} > 1$ [31], the rate $\tilde{R} > f_0$ is typically larger than the threshold rate $R^* \sim ug$ of phenotypic interference. Because the models are defined in the infinite-sites framework [3, 28], no direct balance of genomic costs and benefits of recombination has been performed. However, we expect this balance to differ substantially from the case of phenotypic interference, because the benefit sets in at a larger gene number $g_{\rm m}$ and the cost is determined by a larger threshold rate $\tilde{R}$.

In summary, phenotypic interference generates a marginal cost and a strong benefit of recombination under minimal assumptions on the genome-wide selection and mutation landscapes. These include diminishing return epistasis acting on individual molecular biophysical traits; whereas direct benefits, genome-wide epistasis, and continual adaptation are not required.

Supplementary References

[1] Nourmohammad, A., Schiffels, S. & Lässig, M. Evolution of molecular phenotypes under stabilizing selection. *J Stat Mech Theor Exp* **2013**, P01012 (2013).

[2] Neher, R. A., Kessinger, T. A. & Shraiman, B. I. Coalescence and genetic diversity in sexual populations under selection. *Proc Natl Acad Sci U S A* **110**, 15836–15841 (2013).

[3] Neher, R. A. Genetic draft, selective interference, and population genetics of rapid adaptation. *Annu Rev Ecol Evol Syst* **44**, 195–215 (2013).

[4] Neher, R. A. & Hallatschek, O. Genealogies of rapidly adapting populations. *Proc Natl Acad Sci U S A* **110**, 437–442 (2013).

[5] Hochstrasser, M. Ubiquitin-dependent protein degradation. *Annu Rev Genet* **30**, 405–439 (1996).

[6] Phillips, R., Kondev, J., Theriot, J. & Orme, N. *Physical Biology of the Cell* (Garland Science, 2013).

[7] Monod, J., Wyman, J. & Changeux, J.-P. On the nature of allosteric transitions: A plausible model. *J Mol Biol* **12**, 88–118 (1965).

[8] Manhart, M. & Morozov, A. V. Protein folding and binding can emerge as evolutionary spandrels through structural coupling. *Proc Natl Acad Sci U S A* **112**, 1797–1802 (2015).

[9] Chéron, N., Serohijos, A. W. R., Choi, J.-M. & Shakhnovich, E. I. Evolutionary dynamics of viral escape under antibodies stress: A biophysical model. *Protein Sci* **25**, 1332–1340 (2016).

[10] Otwinowski, J. Biophysical inference of epistasis and the effects of mutations on protein stability and function. *Mol Biol Evol* **35**, 2345–2354 (2018).

[11] Lande, R. Natural selection and random genetic drift in phenotypic evolution. *Evolution* **30(2)**, 314–334 (1976).

[12] de Vladar, H. P. & Barton, N. Stability and response of polygenic traits to stabilizing selection and mutation. *Genetics* **197**, 749–767 (2014).

[13] Nourmohammad, A., Held, T. & Lässig, M. Universality and predictability in molecular quantitative genetics. *Curr Opin Genet Dev* **23**, 684–693 (2013).

[14] Michod, R. & Levin, B. (eds.) *The evolution of sex* (Sinauer Press, Sunderland, Massachusettes, 1988).

[15] Kondrashov, A. S. Classification of hypotheses on the advantage of amphimixis. *J Hered* **84**, 372–387 (1993).

[16] Hartfield, M. & Keightley, P. D. Current hypotheses for the evolution of sex and recombination. *Integr Zool* **7**, 192–209 (2012).

[17] Hamilton, W. D. Sex versus non-sex versus parasite. *Oikos* **35**, 282–290 (1980).

[18] Bell, G. *The Masterpiece of Nature: The Evolution and Genetics of Sexuality* (Croom Helm, London, 1982).

[19] Kouyos, R. D., Silander, O. K. & Bonhoeffer, S. Epistasis between deleterious mutations and the evolution of recombination. *Trends Ecol Evol* **22**, 308–315 (2007).

[20] Salathé, M., Kouyos, R. D. & Bonhoeffer, S. The state of affairs in the kingdom of the red queen. *Trends Ecol Evol* **23**, 439–445 (2008).

[21] Kondrashov, A. S. Selection against harmful mutations in large sexual and asexual populations. *Genet Res* **40**, 325–332 (1982).

[22] Kimura, M. & Maruyama, T. The mutational load with epistatic gene interactions in fitness. *Genetics* **54**, 1337–1351 (1966).

[23] Neher, R. A. & Shraiman, B. I. Competition between recombination and epistasis can cause a transition from allele to genotype selection. *Proc Natl Acad Sci U S A* **106**, 6866–6871 (2009).

[24] Salathé, M., Kouyos, R. D. & Bonhoeffer, S. On the causes of selection for recombination underlying the red queen hypothesis. *Am Nat* **174**, S31–S42 (2009).

[25] Rouzine, I. M., Brunet, É. & Wilke, C. O. The traveling-wave approach to asexual evolution: Muller's ratchet and speed of adaptation. *Theor Popul Biol* **73**, 24–46 (2008).

[26] Jain, K. Loss of least-loaded class in asexual populations due to drift and epistasis. *Genetics* **179**, 2125–2134 (2008).

[27] Goyal, S. *et al.* Dynamic mutation–selection balance as an evolutionary attractor. *Genetics* **191**, 1309–1319 (2012).

[28] Neher, R. A., Shraiman, B. I. & Fisher, D. S. Rate of adaptation in large sexual populations. *Genetics* **184**, 467–481 (2010).

[29] Muller, H. J. The relation of recombination to mutational advance. *Mutat Res* **106**, 2–9 (1964).

[30] Felsenstein, J. The evolutionary advantage of recombination. *Genetics* **78**, 737–756 (1974).

[31] Desai, M. M. & Fisher, D. S. Beneficial mutation–selection balance and the effect of linkage on positive selection. *Genetics* **176**, 1759–1798 (2007).

[32] Tsimring, L. S., Levine, H. & Kessler, D. A. Rna virus evolution via a fitness-space model. *Phys Rev Lett* **76**, 4440–4443 (1996).

[33] Hallatschek, O. The noisy edge of traveling waves. *Proc Natl Acad Sci U S A* **108**, 1783–1787 (2011).

[34] Good, B. H., Rouzine, I. M., Balick, D. J., Hallatschek, O. & Desai, M. M. Distribution of fixed beneficial mutations and the rate of adaptation in asexual populations. *Proc Natl Acad Sci U S A* **109**, 4950–4955 (2012).

[35] Lynch, M. *et al.* A genome-wide view of the spectrum of spontaneous mutations in yeast. *Proc Natl Acad Sci U S A* **105**, 9272–9277 (2008).

[36] Keightley, P. D., Ness, R. W., Halligan, D. L. & Haddrill, P. R. Estimation of the spontaneous mutation rate per nucleotide site in a drosophila melanogaster full-sib family. *Genetics* **196**, 313–320 (2014).

[37] Ossowski, S. *et al.* The rate and molecular spectrum of spontaneous mutations in *Arabidopsis thaliana.* *Science* **327**, 92–94 (2010).

[38] Harrison, P. M., Milburn, D., Zhang, Z., Bertone, P. & Gerstein, M. Identification of pseudogenes in the *Drosophila melanogaster* genome. *Nucleic Acids Res* **31**, 1033–1037 (2003).

[39] Derelle, E. *et al.* Genome analysis of the smallest free-living eukaryote *Ostreococcus tauri* unveils many unique features. *Proc Natl Acad Sci U S A* **103**, 11647–11652 (2006).

[40] Feng, J., Naiman, D. Q. & Cooper, B. Coding DNA repeated throughout intergenic regions of the *Arabidopsis thaliana* genome: evolutionary footprints of RNA silencing. *Mol Biosyst* **5**, 1679–1687 (2009).

[41] Ruderfer, D. M., Pratt, S. C., Seidel, H. S. & Kruglyak, L. Population genomic analysis of outcrossing and recombination in yeast. *Nat Genet* **38**, 1077–1081 (2006).

[42] Fiston-Lavier, A.-S., Singh, N. D., Lipatov, M. & Petrov, D. A. *Drosophila melanogaster* recombination rate calculator. *Gene* **463**, 18–20 (2010).

[43] Comeron, J. M., Ratnappan, R. & Bailin, S. The many landscapes of recombination in *Drosophila melanogaster*. *PLoS Genet* **8**, 1–21 (2012).

[44] Salomé, P. A. *et al.* The recombination landscape in *Arabidopsis thaliana* F_2 populations. *Heredity* **108**, 447–455 (2012).

Supplementary Figures

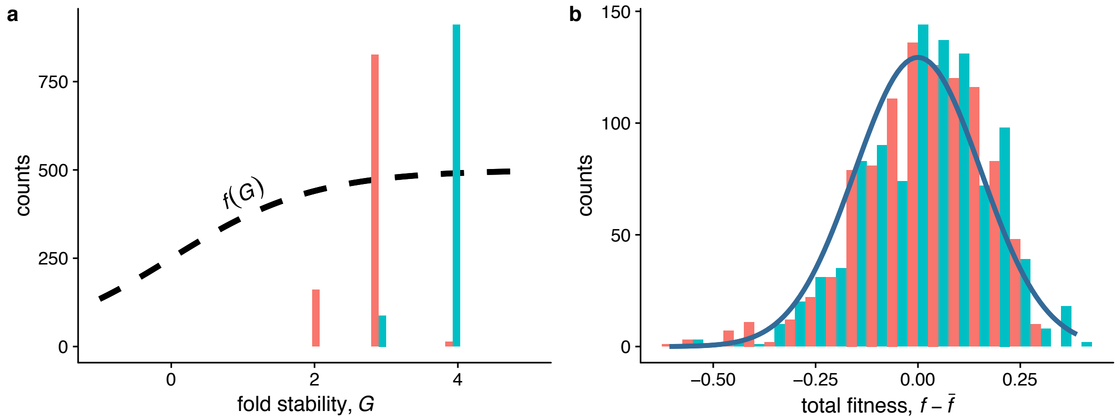

Supplementary Figure 1: Local and global statistics of phenotypic interference. a Distribution of a single protein stability trait G. Snapshots of an evolving population at two uncorrelated time points (marked by blue and orange colors) show strongly peaked distributions with large fluctuations, indicative of a low-mutation regime with strong genetic drift and draft [1]. **b** Distribution of the global fitness f, relative to the mean population fitness $\bar{f}$. Snapshots of the combined fitness variation of all protein traits show a steady fitness wave of approximately Gaussian form with noisy tails [2, 4, 25, 31–34]. Stability is given in units of $k_b T$ (see text); fitness differences are measured per generation. Local and global population statistics are analytically compared in Methods. Simulation parameters: $N = 1000, g = 1000, u = 1.25 \times 10^{-3}$, $\epsilon_G / k_\mathrm{B} T = 1$; see Methods for simulation details.

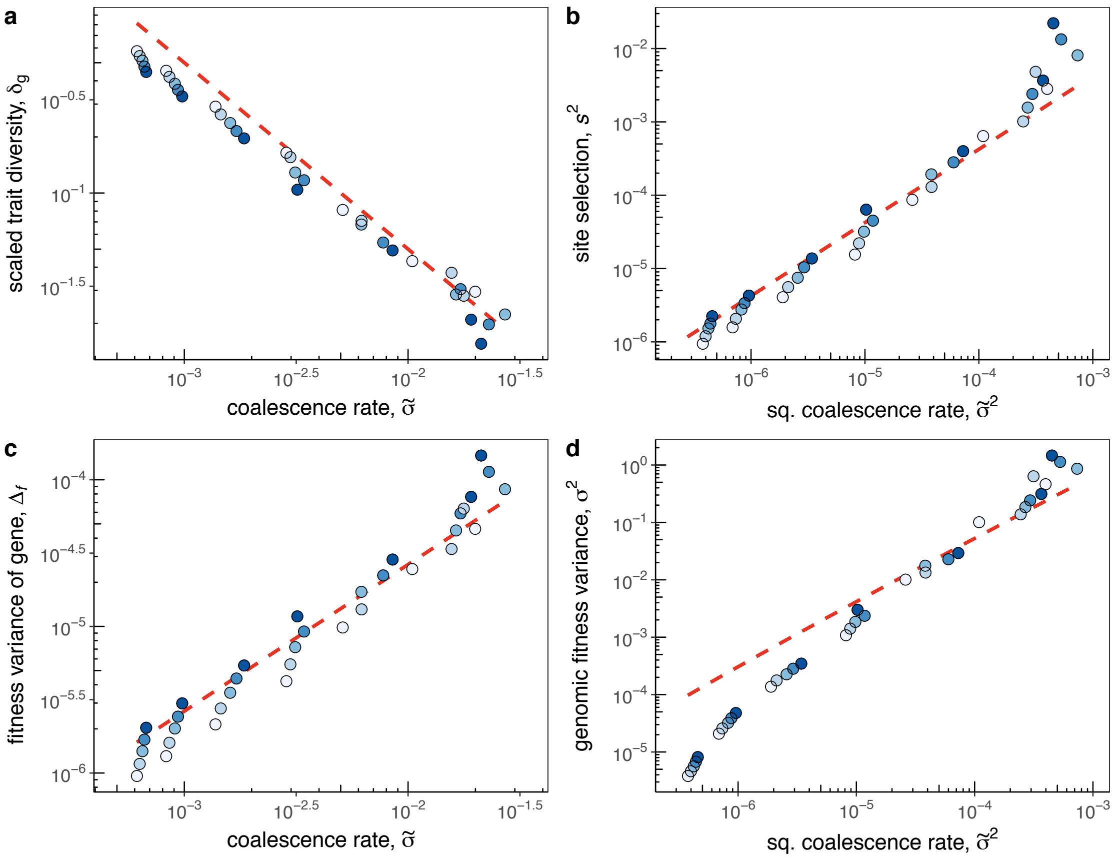

Supplementary Figure 2: Trait and fitness statistics depend on the coalescence rate. Simulation data (dots) for different numbers of genes g and gene selection f_0 (indicated by color) are plotted against the measured (squared) coalescence rate, together with model results under phenotypic interference (red dashed lines). See Fig. 3 for the dependence of these observables on gene number. **a** Expected scaled diversity, $\delta_G = \Delta_G/\epsilon_G^2$, of a single-protein quantitative trait G; phenotypic interference scaling $\delta_g \approx u/(2\tilde{\sigma})$ (red line) as given by Equation (1). **b** Mean square selection coefficient at sequence sites, s^2; phenotypic interference scaling $s^2 = 4\tilde{\sigma}^2$ (red line) as given by Equation (3). **c** Fitness variance per gene, Δ_f; phenotypic interference scaling $\Delta_f = 2u\tilde{\sigma}$ (red line) as given by Equation (3). **d** Total genomic fitness variance σ^2; generic fitness wave scaling $\sigma^2/\tilde{\sigma}^2 = c_0 \log(N\sigma)$, as given by Equation (7) [2, 4].

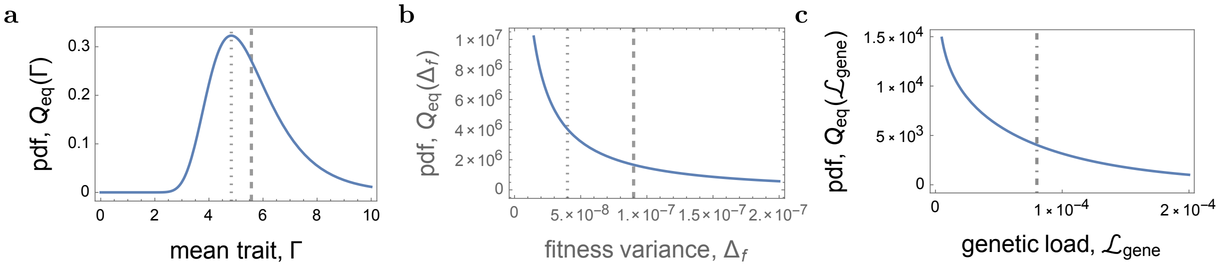

Supplementary Figure 3: Equilibrium distributions under stochastic evolution. The figure shows the probability density functions **a** of the mean population trait, $Q_{\text{eq}}(\Gamma)$, **b** of the conditional expected fitness variance, $Q_{\text{eq}}(\Delta_f)$, and **c** of the genetic load per gene, $Q_{\text{eq}}(\mathcal{L}_{\text{gene}})$; see Equation (S2) – Equation (S6). These distributions measure deviations from long-term averages (dashed lines), which are generated by genetic drift and draft. The corresponding deterministic solutions are marked by dotted lines; both lines coincide in **c**. All pdfs are shown for $\tilde{\sigma} = f_0/100 = 10^{-4}$; other parameters as in Fig. 2. See Supplementary Methods 1.

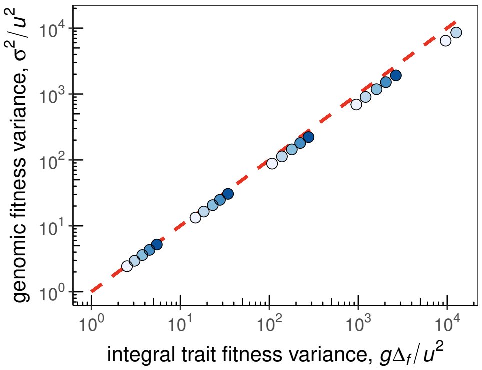

Supplementary Figure 4: Additivity of the genomic fitness variance. For housekeeping evolution in the minimal biophysical model, we plot the total fitness variance, σ^2, against the additive part $\Delta_{f,1} + \cdots + \Delta_{f,g}$. The additivity is used in the closure of the evolutionary dynamics; see Equation (5) – Equation (7) and Fig. 2.

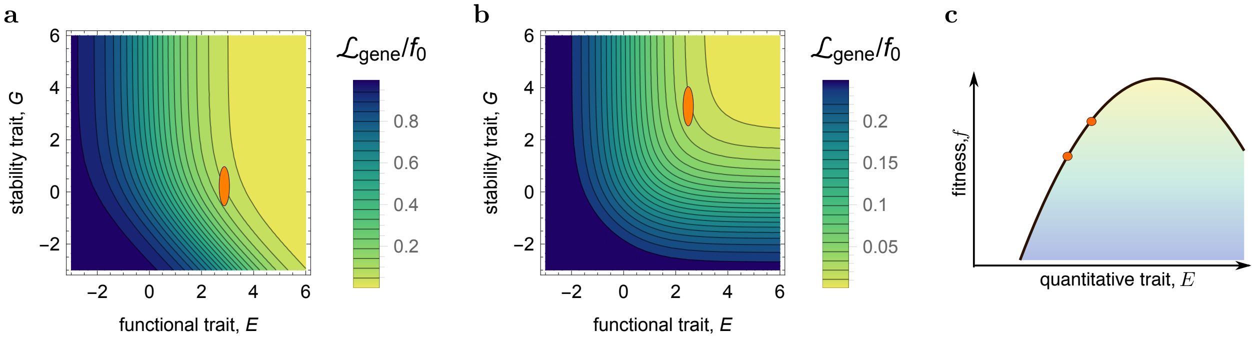

Supplementary Figure 5: Fitness landscapes of extended models. a, b Thermodynamic fitness landscapes $f(G, E)$ of the stability-affinity model, Supplementary Equation S12 – Supplementary Equation S14, are shown as functions of the stability G and the affinity E. Stable populations, characterized by stationary mean values (Γ_G, Γ_E) and variances (Δ_G, Δ_E), are marked by red ellipsoids. **a** Thermodynamic equilibrium. **b** Non-equilibrium driven by active degradation of folded proteins. In the high-fitness part, this landscape becomes approximately additive in G and E. **c** Quadratic fitness landscape $f(E)$, Supplementary Equation S20, as a minimal model for stabilizing selection on a quantitative trait E. Stable population states on a flank of the landscape are marked by red dots. See Supplementary Methods 3; cf. Fig. 1a for the fitness landscape of the minimal model.

Supplementary Tables

	name	description
parameters	u	mutation rate per gene
	g	number of genes
	ϵ_G^2	mean squared mutational effect
	$f(G)$	fitness landscape of single gene (growth rate contribution)
	f_0	selection strength
gene variables	$\Gamma = \overline{G}$	mean trait
	$\Delta_G = \overline{G^2} - \overline{G}^2$	heritable trait variance
	$\mathcal{L} = f_{max} - \overline{f}$	genetic load
	$\Delta_f = \overline{f^2} - \overline{f}^2 \approx \Delta_G f'(\Gamma)$	fitness variance
	$s^2 = \epsilon_G^2 f'^2(\Gamma)$	mean squared selection coefficient
genome variables	$\sigma^2 = g\Delta_f$	global fitness variance
	$\tilde{\sigma} = 1/(2N_\mathrm{e}) = \sigma/\sqrt{c}$	coalescence rate
	$c \approx 10^2$	wave complexity
	$\hat{\sigma}$	tail distance of fitness distribution
	$g_0 = c/4$	min. genome size for phenotypic interference
	R	genome wide recombination rate between genes
	$\xi = g\tilde{\sigma}/R$	linkage block size

Supplementary Table 1: List of mathematical symbols. Parameters are model inputs, gene variables refer to local traits, and genome variables are global at the level of the entire genome.

	Saccharomyces cerevisiae	*Drosophila melanogaster*	*Arabidopsis thaliana*
μ	$3{\cdot}10^{-8}$ [35]	$3{\cdot}10^{-9}$ [36]	$7{\cdot}10^{-9}$ [37]
ℓ	1401 [38]	1500 [38]	2232 [39]
g	6563 [39]	14332 [38]	26990 [40]
R	$3{\cdot}10^{-2}$ [41]	$2{\cdot}10^{-3} - 1{\cdot}10^{-0}$ [42,43]	$2{\cdot}10^{-0}$ [44]
R^*	$6{\cdot}10^{-5}$	$1{\cdot}10^{-3}$	$9{\cdot}10^{-3}$

Supplementary Table 2: Genome data and estimates of threshold recombination rates. Point mutation rate μ, average gene length ℓ (in bp), gene number g and recombination rate R per genome (map length) are shown for three recombining species. The parameter range for *D. melanogaster* describes local recombination rates in different parts of the chromosomes (in the same units) [43]. An upper bond of the threshold recombination rate R^* marking the transition to sexual evolution is obtained from Equation (12) (with $ug = \mu\ell g$ and $c \approx c_0 \approx 100$), cf. Fig. 5a.
