# Supplementary material for: Survival of the simplest in microbial evolution
Source: Nat Commun. 2019 Jun 6;10:2472. doi: 10.1038/s41467-019-10413-8 (PMC6554311; doi:10.1038/s41467-019-10413-8)
Supplement: Supplementary file 3 — Description of Additional Supplementary Files [file 41467_2019_10413_MOESM3_ESM.pdf]

Description of Additional Supplementary Files

File Name: Supplementary Software 1
Description: Computer code for Wright-Fisher simulations.
